# An improved model for provision of rural community-based health rehabilitation services in Vhembe District, Limpopo Province of South Africa

**DOI:** 10.4102/phcfm.v8i2.980

**Published:** 2016-03-30

**Authors:** Rudzani E. Luruli, Vhonani O. Netshandama, Joseph Francis

**Affiliations:** 1 Institute for Rural Development, School of Agriculture, University of Venda, South Africa; 2 Community Engagement, University of Venda, South Africa

## Abstract

**Background:**

In 1991, Riakona Community Rehabilitation Programme initiated community-based rehabilitation (CBR) in the Vhembe District of Limpopo Province. Subsequently, the South African government adopted the programme.

**Aim:**

The aim of the study was to suggest an improvement in the model of providing CBR services.

**Setting:**

The study was conducted in six rehabilitation centres located in hospitals in the Vhembe District in Limpopo Province of South Africa.

**Method:**

A mixed-mode research design with qualitative and quantitative elements was used to conduct the study. Content analysis, the chi-square test for Goodness of Fit and the Kruskal–Wallis and Mann–Whitney non-parametric tests were conducted.

**Results:**

The key determinants of client satisfaction with the services that the community rehabilitation workers rendered included provision of assistive devices and the adoption of a holistic approach to their work. Overall, satisfaction per domain for each one of the five domains of satisfaction scored less than 90%. More than 80% of clients were satisfied with empathy (83%) and assurance (80%) domains. Tangibles, reliability and responsiveness domains had scores of 78%, 72% and 67%, respectively. These results, together with the reasoning map of conceptual framework description, were used as the building blocks of the CBR model.

**Conclusion:**

The improved CBR model is useful for putting the programme into practice. This is particularly so for the CBR managers in the districts of the Limpopo Province.

## Introduction

Measuring client satisfaction is an integral component of hospital management strategies.^1^ Although literature on this subject exists, an audit of Community-based Rehabilitation (CBR) programme methodological research undertaken in the world reveals that there is limited published information on client satisfaction with services that community rehabilitation workers (CRWs) render.^2^ According to Byford et al.,^2^ CBR is frequently implemented in resource-poor contexts, which limits the scope for research on client satisfaction. All evaluations of CBR did not attempt to establish the extent to which clients were satisfied with the services that were provided. Client satisfaction is a highly client-centred indicator of outcome. This means that only the clients can perceive and report their satisfaction.

In the past, health services ascribed to the Medical Model, which prescribed services to all its customers. Adoption of a more social model has now seen provision of healthcare services increasingly recognising the rights of clients. However, a client-centred approach requires legislation before the management of healthcare centres takes cognisance of the views of clients.^1^

Research has shown that clients who are satisfied are more likely to remain with a health worker, continue to use the services a medical centre provides and refer other patients to the latter.^3,4^ Clients who are satisfied keep appointments and are also more likely to comply with treatment.^4^

Client satisfaction tools are by their very nature instruments that even when used properly can only assist in obtaining an impression of the client’s satisfaction with the service rendered. Moreover, quantitative studies of this nature only measure the items in the questionnaire. In contrast, qualitative methods provide a richer and deeper understanding of what clients think. Thus, it is not surprising that the Luruli^5^ study recommended the use of the client satisfaction survey tool in combination with qualitative methods. Therefore, the study reported in the article adopted this recommendation. Using the client satisfaction tools that Luruli^5^ developed, modifications were made and used to evaluate client satisfaction. Specifically, an adapted version of the SERVQUAL framework (Parasuraman et al.)^6^ was used.

**FIGURE 1:**
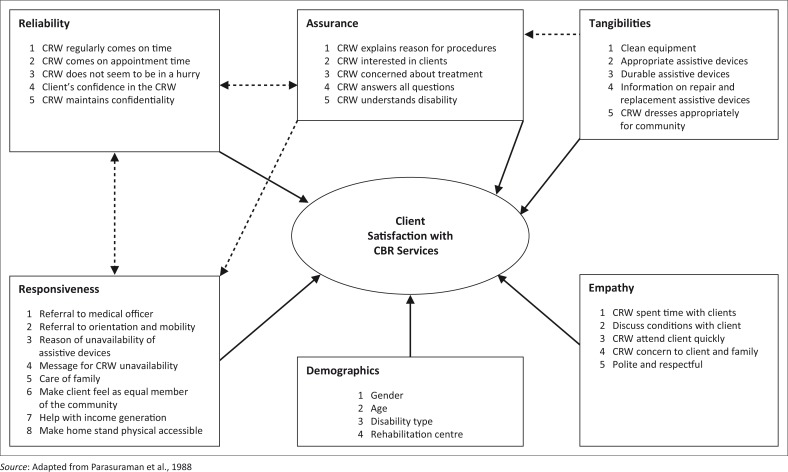
Conceptual framework of evaluation tool for client satisfaction with community-based rehabilitation services.

Sociodemographics were also considered as important predictors of client satisfaction. These included clients’ sex, age, type of disability and rehabilitation centre through which services were rendered. The adapted conceptual model for the client satisfaction evaluation is presented as Figure 1. The SERVQUAL instrument is the theory that underpins this conceptual framework used for assessing customer perception of the conceptualisation and operationalisation of the service quality constructs. The procedures used to construct and refine a multiple-item scale to measure the construct are described. Overall, the purpose of this framework is to develop the domain of the service quality, which leads to client satisfaction. The scale that is the focus of this framework involves perceived quality, which is the consumer’s judgement about the entity’s overall excellence.^7^

Various studies were conducted to find out if the domains of client satisfaction statistically differed because of gender. Mixed results were obtained in which there was confirmation and also rejection of findings of previous studies. For example, the results of the study by Roohi and Asayesh^8^ were inconsistent with these findings because a significant gender effect was observed for the empathy domain. In the current study, 52% of the clients were female participants, whilst in the study by Roohi and Asayesh^8^, the proportion of female participants was 89.7%. Also, in the current study, significant gender effect was not observed for the reliability domain, although it was observed in the study by Mokhlis et al.^9^ In the study by Janikowski and Bordieri^10^ and Rast and Tourani,^11^ no significant differences were found across all the five domains because of gender, which were contrary to the study by Yunus et al.^12^ According to Filmer,^13^ women with disabilities face discrimination based on both their gender and disability. Thus, any CBR programme should promote and support gender equality and empowerment of women, in particular.

Available literature points towards the fact that another defining factor regarding client satisfaction with CBR services is age of the beneficiary. The studies by Roohi and Asayesh^8^ and Mokhlis et al.^9^ revealed that the age of the client significantly influenced the empathy, reliability, assurance and tangible domains. The current study went further and pointed out that there were statistically significant age differences in client satisfaction perceptions with respect to the responsiveness of CRWs.^8,14^ The study that Janikowski and Bordieri^10^ undertook did not reveal any significant difference in perceptions across all the five domains because of age. It can be argued that the differences observed above emanated from the fact that the settings were varied in terms of country, cultural and other factors.

Some studies have also tested the existence of statistically significant differences of client satisfaction per domain because of types of disability. Chappell and Johannsmeir^15^ found that differences caused by types of disability existed. This finding confirmed that diverse opinions existed across the types of disability with regard to satisfaction with CBR services. The latter studies did not make use of domains of client satisfaction. Instead, focus groups were constituted to deliberate on client satisfaction in general. However, the results could be classified under the empathy, reliability and assurance domains. They revealed that interventions of community rehabilitation facilitators across various types of disabilities were highly important.

Mwansa^16^ investigated the satisfaction of persons with disabilities regarding services received at four primary healthcare centres in Ndola, Zambia. Statistically significant differences in the perception of level of satisfaction with availability of healthcare services amongst persons with disabilities and also rehabilitation centres were observed. The current study was conducted in six rehabilitation centres of Vhembe District and confirmed the findings of the latter study in Zambia. However, because of the lack of consensus around the concept of CBR, local conditions and agreed principles of CBR were taken into account before client satisfaction was measured, and the results were used to develop the model for provision of CBR services.

## Purpose of the study

This study was carried out with the aim of investigating client satisfaction with respect to the provision of rural community-based health rehabilitation services in order to develop an improved model for CBR programme in the Vhembe District, Limpopo Province.

## Objectives

To suggest an improved model for provision of rural community-based health rehabilitation services provided by CRWs of Vhembe District, Limpopo Province.

## Research methods and design

### Study design

A mixed-mode research design that took advantage of the complementary attributes of qualitative and quantitative elements was used. Also, the study was exploratory and descriptive in nature. Phase 1 focused on in-depth interviews and focus-group discussions. In phase 2, an adapted questionnaire containing closed-ended questions on client satisfaction was administered.

The experiences of people with disability regarding CBR services were explored and compared with relevant literature. This formed the basis of identifying and conceptualising the concept following Creswell^17^ eight steps of thematic content analysis. The framework of Dickoff and James^18^ was used to describe and develop the model.

During the first phase in which qualitative data were collected, the experiences of clients of CBR services were explored through in-depth individual interviews and focus-group discussions. The central question provided direction in the interviews. Questions that emanated from the discussions were also explored further using the probing technique of interviewing. Data were analysed following the eight steps that Creswell^17^ recommended. Distinct themes, categories and sub-categories that emerged were pulled out. Dense descriptions of these experiences were also provided in the light of relevant literature.

In phase 2, quantitative data were collected in order to determine whether CRWs were empathic, reliable, assuring, responsiveness and produced tangible results when serving their clients. This involved developing a questionnaire for client satisfaction services. The questionnaire was piloted with 19 respondents and subsequently administered to 357 clients.

### Setting

The study was conducted in six rehabilitation centres located in hospitals located in the Vhembe District. The focus on CBR programme was even more important because of the development of primary healthcare programme in the district health system.

Vhembe is one of the five districts in Limpopo Province. Limpopo is ranked as the second poorest Province in South Africa. The study area is a rural district which was part of the former Venda homeland that the Apartheid system created. It is regarded as the second poorest district in the Province. There are six hospitals in the Vhembe District where 19 CRWs serving rural communities operated from.

### Study population and sampling strategy

The study population consisted of all the 2850 disabled clients that the CRWs in Vhembe District worked with. Within this population were persons with four types of disability, namely, visually impaired, physically challenged, having speech and hearing difficulties and mentally impaired. For the quantitative study, 357 respondents participated in the survey. Lastly, a qualitative study of 10 people was carried out. The latter entailed carrying out in-depth one-on-one interviews. A focus-group interview involving 15 participants was also conducted in an attempt to enrich the data through triangulation.

### Data analysis

Thematic content analysis was used to draw meaning from qualitative data. With respect to the quantitative study, reliability and validity were achieved using the Cronbach’s alpha technique. The Statistical Package for Social Sciences version 22.0 was used to analyse the quantitative data. Cross-tabulations were computed. The chi-square test for Goodness of Fit was carried out together with the Kruskal-Wallis and Mann-Whitney tests. This was based on the realisation that the non-parametric data collected did not follow a normal distribution. The tests were conducted to determine whether there were differences in empathy, reliability, assurance, tangible and responsiveness because of gender, age, type of disability and community rehabilitation centre. The equation that guided the data analyses is presented in Box 1.

**BOX 1:** Data analyses.

Level of client satisfaction	=	*f*(gender, age, type of disability, community rehabilitation centre) + random error

*Source*: Own primary research data

*f* = type of domain of satisfaction.

The hypotheses tested in the study are as given below:

- **H_0_: 1:** There are no differences in the client-perceived levels of satisfaction with regard to empathy, reliability, responsiveness, assurance and tangible across disabilities.
- **H_a_: 2:** The levels of client satisfaction in terms of empathy, reliability, assurance, tangible and responsiveness within each type of disability are the same.

## Ethical considerations

Ethical approval was secured from the University of Venda’s Research Ethics Committee and also the Department of Health and Social Development Ethics Committee. All the protocols that guide scientific research in the social sciences were adhered to. Amongst these were informed consent, anonymity, confidentiality, privacy, no harm to respondents and the principle of voluntary participation and beneficence. Written or verbal consent was obtained from the respondents. The parent/mother or caregiver gave informed consent on behalf of children and the mentally challenged participants.

## Results

The qualitative study revealed that clients had unique experiences regarding services that CRWs in the Vhembe District rendered. Five themes and associated sub-themes were drawn. The experiences related to extent of satisfaction with services such as provision of assistive devices, guidance achieved towards positive lifestyles, the holistic approach used, nature of care provided and significance of health education offered. It was also revealed that the assistive devices provided were of poor quality, there were community barriers towards service rendering, promises were not fulfilled to satisfaction, CRWs visited clients infrequently and the CRWs were regarded as untrustworthy. Some positive aspects of service were highlighted. They included good interpersonal relationships that existed with some CRWs, accommodation of client’s different problems, prompt response to clients’ needs, encouragement of positive attitude or behaviour and strengthening of trustful relationships. In order to improve the provision of CBR services, the clients proposed more frequent, reliable CRW visits to clients, provision of special assistive devices, making available reliable transport for CRWs and introduction of income-generating community projects for the disabled, which were necessary interventions. It was evident that the interventions should be central to the proposed model for improvement of the provision of CBR services.

Statistically significant relationships were observed between the availability of healthcare services and level of satisfaction of persons with disabilities across the rehabilitation centres and all the five domains. The average scores for all the domains of client satisfaction varied from 67% to 83%. However, this should not result in health managers being overly gratified that their clients were satisfied with the way the CBR services were rendered. It was particularly discomforting to find that the score for the responsiveness domain was the lowest amongst the five. It was found that ‘sending a message that he or she would not be available’, which a perceptions of responsiveness, scored the lowest in comparison to the others.

Each of the five domains was further explored on its own with a view to isolate and highlight any interesting patterns that emerged. In general, the results obtained for all the domains of service delivery were scored less than 90%, which signified client dissatisfaction. This was not surprising considering the fact that rehabilitation in Limpopo Province was not yet integrated into the primary healthcare system.

Client satisfaction tools are, by their very nature, commonly quantitative instruments that even when used properly can only assist in obtaining an impression of the client’s feelings about the service. Such quantitative studies only measure the items in the questionnaire. In the current study, the significance of effects of determinants of perception on client satisfaction because of gender, age group, type of disability and rehabilitation centre were tested. The results are covered under empathy, reliability, assurance, tangible and responsiveness domains as shown in Table 1.

**TABLE 1:** Significance of effects of determinants of perception on client satisfaction.

Domain of client satisfaction	Descriptors of the domain of satisfaction	Overall satisfaction per domain %	Significance of effects observed for any descriptor of the domain of client satisfaction			

			Gender	Age group	Type of disability	Rehabilitation centre
Empathy	1. The CRW spent time talking to you whenever you need him/her.	80	No	No	No	Yes
	2. The CRW discussed your medical condition with you.	87	No	No	No	Yes
	3. The CRW attended to you quickly when you asked for help.	67	No	No	No	Yes
	4. The CRW was concerned when speaking to you and your family.	86	No	No	No	Yes
	5. The CRW was polite and showing respect when speaking to you and your family.	94	No	No	No	Yes
Reliability	1. The CRW always come on the day he/she promised to visit you.	51	Yes	No	No	Yes
	2. The CRW came on time for the appointment.	51	Yes	No	No	Yes
	3. The CRW did not seem to be in a hurry when he/she was with you.	84	No	No	No	Yes
	4. You had enough confidence in the CRW to discuss personal issues or problems.	85	No	No	No	Yes
	5. The CRW maintained your confidentiality at all times.	88	No	No	No	Yes
Assurance	1. The CRW explained to you the reason for procedures which were carried out on you.	80	Yes	No	No	Yes
	2. The CRW took an interest in you as a person and not just your condition/disability.	84	No	No	No	Yes
	3. The CRW spent time with you discussing your concerns regarding your treatment.	82	No	No	No	Yes
	4. The CRW tried his/her best to answer all your questions.	74	No	No	No	Yes
	5. You had a clear understanding of your condition/disability during the time you received service from the CRW.	82	No	No	No	Yes
Tangible	1. The equipment for assessment or treatment brought by the CRW was clean.	86	No	No	Yes	Yes
	2. The CRW provided assistive devices that were appropriate to your community.	69	No	No	Yes	Yes
	3. The CRW provided the assistive devices that were durable.	72	No	No	Yes	Yes
	4. The CRW provided you with information on how to repair or replace assistive devices accessories.	71	No	No	Yes	Yes
	5. The CRW was dressed in a way that was appropriate to your community.	93	No	No	Yes	Yes
	Responsiveness	1. When the CRW could not help you, he/she referred you to the hospital to be seen by the therapist or medical Officer.	84	Yes	No	No	Yes
		2. The CRW refers you to orientation and mobility services.	71	No	Yes	Yes	Yes
		3. When assistive devices are not available, the CRW explains the reasons why they are not available.	64	No	No	Yes	Yes
4. When the CRW could not come for an appointment, he/she sent a message that he/she would not be available.		40	No	No	No	Yes
5. The CRW does care about your family.		81	No	No	No	Yes
6. The CRW makes you feel as equal members of the community.		83	No	No	No	Yes
7. The CRW helps you with income generation ideas.		53	No	No	Yes	Yes
8. The CRW helps you to make your homestead physically accessible.		63	No	No	No	Yes

Source: Own primary research results CRW, community rehabilitation worker.

The infusion of qualitative methods helped provides a richer and deeper understanding of what clients were thinking. However, the case study nature of the study, though incisive and robust, made it difficult to generalise the findings. This was the case despite the fact that using quantitative and qualitative methodologies in combination enhanced the chances of obtaining a more accurate picture of the perceptions of the clients regarding the extent of their satisfaction with provision of CBR services. The results of both qualitative and quantitative studies were then used to propose an improved model for provision of CBR services in Limpopo Province. In the next section, the key features of the improved CBR model are articulated.

Five main themes and associated sub-themes were distilled during data analysis. The main themes were as follows: experiences related to satisfaction with services the CRWs rendered; challenges associated with CBR service provision; positive aspects related to CBR service delivery; clients’ suggestions on how to improve delivery of the services and important interventions for an improved CBR model within the context of rehabilitation processes.

Overall client satisfaction scores for the five domains of client satisfaction were 83% for empathy, 80% for assurance, 78% for tangibles, 72% for reliability and 67% for responsiveness. Statistically significant differences with respect to satisfaction with empathy were observed amongst rehabilitation centres (*P* < 0.001). Significant differences (*P* < 0.001) were observed across rehabilitation centres in perceived client satisfaction with respect to assurance, reliability, tangible and responsiveness domains. Differences because of gender were found to exist in perceived reliability and responsiveness of CRWs. Also, perceived client satisfaction in terms of tangible outcomes and responsiveness of CRWs significantly differed (*P* < 0.01) amongst the types of disability. These results highlighted the need for a context-specific model for improved provision of CBR services.

The major challenges militating against the provision of CBR services in Vhembe District were cited and are listed below. These challenges provided a template to use when constructing the improved model for delivering CBR services:

1. Non-existence of a programme that can be used to upgrade the skills and knowledge of CRWs.
2. Poor management of CBR services in the District office.
3. Lack of integration of the CBR programme within the primary healthcare system.

## Towards a model for improved provision of rural community–based health rehabilitation services

### Agent (Who performs the activity?)

The agents responsible for implementing the CBR model/framework are CRWs and supervising therapists. Their activities are designed to contribute positively towards independent living amongst persons with disabilities. CRWs and therapists are, respectively, classified as primary and secondary agents of the CBR programme. This classification is based on the realisation that CRWs serve as the first contacts with people with disabilities in the communities they operate in. Therapists are secondary agents because they relate with clients through a treatment setup. Moreover, the therapists supervise the CRWs and thus are often regarded as clinical supervisors. Their role is to impart knowledge and skills to the CRWs.

### Patiency or recipient (Who is the recipient of the activity?)

In the current model, patience refers to people with disabilities. The agent and recipient engage each other in a treatment session. This is designed to ensure that the person with disability is empowered to be independent in performing activities of their daily living. For this to work, there is need for people with disabilities to learn to trust CRWs and therapists. This facilitates open discussion of challenges militating against their health and well-being. In turn, the professional workers should demonstrate sufficient honesty and integrity to earn the trust.

### Framework (In what context is the activity performed?)

The context of the model should be viewed on the basis of a matrix of activities. Central to this matrix are persons performing various activities plus the interrelation of these factors because they constitute an organism, unity or total context of implementation of the activity.^18^ Some of common contexts identified within the entire district health system where CBR services are supposed to take place are:

1. the community’s culture, which encompasses shared sociocultural beliefs;
2. family realities, taking into account that members play various roles such as being mothers and care givers, amongst others. Inequalities between people with disabilities and their family members might exist and
3. school status quo based on the fact that disabled children need to be integrated into a normal school for them to receive formal education; some teachers might be reluctant to accept children with disabilities in normal schools
4. district hospitals for referral purpose from health centres and clinics.

### Dynamics (What is the energy source for the activity-whether chemical, physical, biological, mechanical, or psychological, etcetera?)

Dynamics are regarded as the sources of power for CBR activities. They can be in the form chemical, physical, biological or psychological dynamics. The common denominator amongst them is that any person or thing functioning as an agent, patient or part of the framework needs them in order to realise the set goal.^15^ Taking this into account, the dynamics of the proposed CBR model include embedding the services into the integrated primary healthcare strategy of the district or province in which the CRWs and therapists need to exercise professionalism and commitment in partnership with people with disability. The dynamics has the following components: professional role of CRW, supervisors of CRWs and CBR manager as well as commitment in partnership with people with disabilities. The factors listed below influence professionalism and commitment in partnership in the interaction between the main role players in the improved CBR model:

1. **Combating power inequality:** It is most likely that by virtue of CRWs or therapists being people with power, they might assume positions of authority. That is likely to relegate the contributions of the people with disabilities to irrelevance when making decisions.
2. **Honesty:** Both CRWs and their clients should view this as a critical ingredient that guarantees effective communication amongst them.
3. **Openness:** This is closely linked to honesty. Being open during discussions is crucial because areas of (dis)satisfaction are often easily disclosed.
4. **Mutual trust:** Such trust allows participants to take or share advice without suspicion that inhibits progress.

### Procedure (What is the guiding procedure, technique or protocol of the activity?)

The guiding procedure in the current model is that CBR services should be implemented as part of an integrated primary healthcare strategy. This is why in this model the roles of the main implementers of the CBR programme have been explicitly articulated. In this regard, the roles of CRWs, therapists and manager are explained below.

### Terminus/purpose (What is the end point of the activity?)

Terminus refers to the end point or accomplishment of one or many activities.^15^ Improvement of the quality of life of people with disabilities is the terminus of the improved CBR model. Central to all this is the quest for empowering the clients so that they do things for themselves.

## Development structure of the community-based rehabilitation model

The Dickoff and James^18^ model was adapted in the study in order to reflect a conceptual framework that supports the development of the CBR programme. For this reason, the conceptual framework was a reasoning map that mainly focused on the survey list of Theory in a Practice Discipline. It encapsulates the agent and recipient of the CBR service, context of operation, procedures followed, dynamics that must be addressed and lastly the terminus.^18^

Dickhoff et al.^18^ contend that the survey list should respond to the six crucial questions about the activities to be performed. The survey lists in the current model are agent, referring to CRWs; recipient (people with disabilities who need rehabilitation); context (the CBR programme in district healthcare systems); dynamics (CBR services in the integrated primary healthcare strategy); procedure (guiding steps or approach for implementation of CBR programme) and terminus (improvement of life of people with disabilities). It must be noted that the answers to the questions should have interactive significance to one another as implementation unfolds. Key dimensions of the model are further clarified below.

### Community rehabilitation as an agent

Successful implementation of the improved CBR programme depends on the calibre of CRWs who are the frontline agents. The CRWs are health workers who should be mid-level professionals registered with the Health Professions Council of South Africa. Such professionals are expected to be knowledgeable, appropriately skilled in providing CBR services, enthusiastic, have constructive attitudes, confident, committed, effective in communication, compassionate, resourceful, well organised and ethical in their professional conduct. It is also expected that the CRWs will have appreciable personal qualities and possess empowering skills such as the ability to provide reliable information when required. Their interpersonal relationship skills (to listen and show empathy) as agents of the CBR programme must be impeccable.

### The role of people with impairment or with disabilities in executing their individual programmes

Empowerment of the people with impairment or disabilities will be in the form of imparting skills and providing relevant information required to develop strategies to deal with constraints encountered in the course of managing their home programmes. People with disabilities who need rehabilitation should manifest impairment that affects roles of life; activity limitation (open opportunities to participate in support groups for better interpersonal relationships) and community integration roles (provide support to motivate participation in income generation projects through support groups). The latter should include communication skills that enable them to bargain and negotiate. At district level, the CBR programme should be integrated into the district health system. Such a system should contain legal, ethical and professional frameworks. Furthermore, there should be capacity to manage resources and provide required support. Adequate policies should be in place, in addition to having effective systems for monitoring and evaluating the implementation of the CBR work.

### Community-based rehabilitation in District health system (framework/context)

#### Community social and cultural contexts

This proposed CBR model is expected to be implemented within the social and cultural contexts of the Venda- and Tsonga-speaking communities in Vhembe District. Linked to this is the infusion of the Dickoff and James^18^ contextual underpinnings as outlined in the third aspect of the survey list of the Theory in a Practice Discipline.

#### Legal ethical and professional framework

The CBR model process is outlined within a clear policy and legal framework. The activities of the CBR programme are executed within the provisions of the following legal frameworks: *Health Professions Act* 56 of 1974; Constitution of the Republic of South Africa of 1996; *Non-profit Organisation Act* of 1997; *Mental Health Care Act* 17 of 2002; *National Health Act* 61 of 2003; *Children’s Amendment Act* No. 38 of 2005 and *Older Persons Act* No.41 of 2007, amongst others.

#### Managing resources and management support

The work environment should provide learning opportunities which will encourage the CRWs to perform to their potential. If this happens, authentic leadership and empowerment of CRWs are likely to be achieved. All this might make the CRWs more committed frontline agents of the CBR programme, devoted to continuous learning and achievement of institutional goals.

### Policies, monitoring and evaluation tools to achieve expected outcomes

Policies and procedures are formulated based on national and provincial realities in order to achieve the expected outcomes, which in this case are enunciated in the National Rehabilitation Policy of 2000. This context should be conducive for the CRWs to execute their assigned responsibilities. The dynamics which are the underlying forces for execution of a CBR programme within the district health system have been explained in other parts of the article.

## Professionalism and commitment in partnership (dynamics)

### Professionalism

Improvement of the life of people with disabilities depends on skills for being a competent client empowered clients who can live independently and autonomy in the execution of life-promoting roles and social integration of client satisfaction with CBR. All these ideals can only be realised if the major players’ roles and responsibilities are clarified. Thus, the guiding procedure for implementing the CBR programme involves clarifying the roles of the CRW, supervisor and manager and also commitment in partnership with people with disabilities.

The CWR is expected to promote an understanding of disability through health promotion. As they execute their tasks, CRWs help people with disabilities to access resources that enhance income generation, attainment of education and acquisition of required skills, empowerment and participation in family and community affairs. The CRWs use rehabilitation activities, exercise and assistive devices appropriate to their community so as to enable this to happen. The CRW can work independently most of the time but there is a need for a therapist to supervise the activities, which under normal circumstances entail support, problem solving, administering assigned tasks, professional behaviour and accountability.

Supervision of CRWs must include support, education and monitoring. During monitoring, the supervisor should help where necessary. They are expected to give constructive advice on all aspects relating to client care, as well as constructive feedback on client records, reports and statistics.

Managers of CBR programmes provide direction, policy and general support for successful delivery of services in the entire district. They must ascertain the operational goals and policies of the CBR unit. This allows the translation of operational goals into quantifiable management objectives. In addition to this, the CBR manager is expected to disseminate information and monitor the attainment of set standards.

#### Commitment in partnership

**Motivation:** Motivation will form as source of encouragement for the client during the CBR services process because they will want to achieve the set goals in order to obtain the results and to know that their efforts contributed towards the effective management of a CBR programme. The client could be motivated through attendance of support group workshop in which they can lead how to keep a good spirit of managing a home programme. Furthermore, the CRWs should encourage the client to participate in income generation project.

**Responsibility and accountability:** The clients will be expected to explain and justify their action during CBR services. Clients should take responsibility for all actions during the execution of daily home programme.

**Interpersonal relationships:** The CRWs and people with disability are responsible to cultivate and maintain positive working relationships in the CBR programme because it results in marked respect, honesty and commitment to the mission of the programme by all members. Clients should maintain positive working relationships with other clients and all stakeholders involved in CBR programme, including the CRW who will be responsible to empower them with the CBR skills.

**Communication skills:** The client should have a negotiation skill that will enable him and/or her to bargain for an aspect that could assist during CBR process that could lead to achievement of set goals. The client will be able to voice out needs that could help in understanding all areas of concern. The CBR programme should accommodate the fact that each individual is unique and that the client has his and/or her own characteristics that should be taken into cognisance during the implementation of the CBR programme. Therefore, the CBR programme should consider the needs of the clients.

### Community-based rehabilitation in an integrated primary healthcare (procedure)

#### Strategies to ensure effective CBR services

Effective strategies are required to stimulate change and development in the CBR programme. The CRWs, clients and framework/context should require an enabling environment for implementation of the CBR programme. Above all and as has been mentioned elsewhere, it is crucial to empower the people with disabilities. That the CRWs need skills and knowledge to execute their work effectively cannot be contested.

#### Values clarification in community-based rehabilitation programme

It is normal that CRWs will encounter various challenges as they implement the CBR programme. Some of the challenges might be a product of differences in values and principles that guide the persons in the forefront of managing the CBR. Such difficulties should be managed in such a way that CRWs and others working with them view these as a motivation to realise the set goals of their programmes.

#### Pre-requisites for effective community-based rehabilitation programme

The underlying dynamics for implementation of a context-specific CBR programme must address the identified problems that CRWs experienced when executing their roles and responsibilities. The interactive facilitation of context-specific CBR programme requires that all stakeholders involved in the CBR management in a district have a shared understanding of its goal and implementation thrust. Central to this is the need for introducing effective trust building and sustenance mechanisms.

### Empowerment of people living with disabilities to live independent life to their potential (Terminus)

The endpoint of the CBR programme is the outcome, which confirms whether set goals for the activity in question were achieved. Improvement of the lives of people with disabilities is the desired end point of the CBR service delivery-related activities. Within the context of the study, successfully rehabilitated clients should enjoy better lives. For this to happen, the following broad objectives should be achieved: to equip clients with skills for them to become competent, to empower persons with disabilities for independent living and to attain autonomy in execution of life-sustaining activities.

### Skills for being competence clients

The clients are expected to successfully implement their individual rehabilitation programme and achieve all set goals for improvement of their life. In order to achieve the set goals, the CRW must have the resources to perform the activities, plan how to use the resources when executing the activity, record all that is accomplished and report on what was performed. Availability of physical, social and spiritual resources enables implementation of planned activities and thus the achievement of set goals.

### Empowered clients leading to independent living

The expected outcome of empowering clients in this model is clients who are satisfied with successful implementation of the CBR programme. It is advisable to ensure that the clients are involved in planning and executing their rehabilitation programme. This creates a sense of ownership and builds self-esteem, which unlocks potential and ideas that might further grow and develop the CBR programme.

### Autonomy in execution of life role

It is imperative for the CBR programme to be context-specific. Also, as already been alluded to, clients should be empowered through equipping them with skills that enable them to be autonomous when managing their own CBR programme. Evidence of the autonomy would include the ability to manage their daily living effectively and enjoy a high quality of life. In this context, autonomy means that clients should be afforded the power to make and execute crucial decisions during the rehabilitation process.

## The proposed structure of the community-based rehabilitation model

**FIGURE 2:**
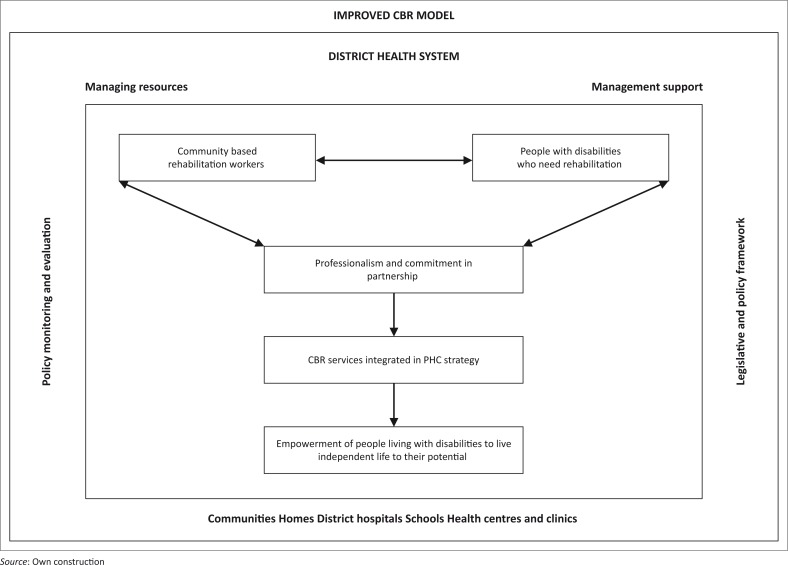
The proposed model for improved provision of community-based rehabilitation services in Limpopo Province.

Figure 2 depicts the outcome of the CBR service model, namely empowerment of people living with disabilities to live independent life to their potential. The model has the potential to facilitate the implementation of the CBR programme. It is process oriented.

In the article, the conceptual framework, which formed the basis of the development of the improved CBR service delivery, model was outlined. The survey list of Theory in a Practice Discipline, which includes agent, recipient, context, procedure and dynamics, informed this process. Lastly, the purpose was adapted and outlined in the context of the study.^15^ The survey list responds to the six crucial questions relating to the activities that must be performed. The answers to the questions should have interactive significance to CRWs, therapists and CBR managers as implementation of the CBR programme proceeds.

## Recommendations

Based on the findings of the study, recommendations for research, policy and practice have been made. There is a need to test the effectiveness of the proposed improved CBR service delivery model. Apart from this, social, political and economic costs and benefits of the CBR service delivery model should be explored. This is necessary in the quest for funding and promotion of a sustainable CBR programme. With respect to practice, the development of a policy brief and guidelines might enhance uptake of the improved CBR model.

## Conclusion

In this article, the studies that served as the building blocks of a model for improved CBR service delivery have been explained. Components of the improved CBR model have been articulated. Its specific components or building blocks are the following:

1. Agents of CBR services are CRWs and therapists.
2. Recipients refer to people with disabilities who need rehabilitation.
3. Infusion of the CBR in the district health system constitutes the framework/context.
4. Dynamics, which represent professionalism and commitment in partnership.
5. Procedure, which is CBR integrated in PHC strategy.
6. Terminus/purpose, which is improvement of life of people with disabilities to live independent life to their potential.

The end point in the CBR programme is that the clients who are rehabilitated should have improved lives that can lead them to being satisfied with services rendered to them. It has also been highlighted that skills for being competent clients, empowered clients enjoying independent lives and demonstrable autonomy in executing activities that shape their lives. It is envisaged that when all these happen, client satisfaction with the community-based rehabilitation programme would be high.
